# The Milk Supply of Hospitals

**Published:** 1907-05-18

**Authors:** 


### The Milk Supply of Hospitals.

Recently a conference of delegates from nine hospitals was held in the board-room of the Hospital for Sick Children, Great Ormond Street, and after discussion various resolutions were passed concerning the milk supply. Put shortly, the conference recommended that the hospital committees shall insist on a supply of pure, genuine, unadulterated milk, from healthy cows which are examined quarterly by a veterinary surgeon; that the milk shall contain at least 3.50 per cent. of butter-fat; that the hospitals shall have the right of inspection of the source of supply at any reasonable time; that the milk shall be strained and refrigerated at the farm, canned and sealed there, and delivered sealed at the hospital; that it shall not be "pasteurised" without the written sanction of the hospital authorities; and that the milk shall be tested bacteriologically and chemically once a week.

All these are excellent and practical recommendations, which ought not to be beyond the executive power of any hospital committee to enforce. Pure, clean, unadulterated, and uncontaminated milk is a crying need, for infants particularly. The difficulty in obtaining it has led to the adoption of methods of dealing with milk which seriously modify its natural properties. This is clearly realised by the inclusion of the clause prohibiting "pasteurisation." It has become the custom of some dairies to treat milk in this way, in order to prevent it "souring" in hot weather and to destroy deleterious organisms. Much can be said against the process. Although it destroys most of the dangerous organisms, many escape and grow even the more readily, because the harmless lactic acid bacillus is also killed. Further, the "souring" of milk is a valuable danger-signal, for it shows at once that the milk is no longer fresh and suitable for infant feeding. Serious chemical changes may take place in "pasteurised" milk without yielding any evidence to taste or smell. Milk so treated, moreover, is of much less value than is uncooked milk for infants in either good health or bad.

The same objections hold good in the case of milk heated to a higher temperature, as in sterilisation. This is one reason why we are strongly opposed to the municipal supply of sterilised milk for infant feeding. Although it is well known that infants may be reared successfully on undiluted sterilised milk, such a food has little, advantage over some of the many proprietary foods in the market. Municipal bodies would be better advised to devote their energies to the provision of pure, clean milk, to the more careful supervision of the sources of the milk supply, to the sanitation of dairies, and to the methods of delivery. We have reached a stage in which it is justifiable to throw upon the municipality the onus of prohibiting the sale of milk, which does not reach certain chemical and bacteriological standards of quality. Until this is done, the hospitals must take special precautions to secure a supply of good milk.

There is no doubt that milk should invariably be strained and refrigerated at the farm, that it should there be canned and sealed, and that it should be delivered sealed. The structure of the milk-can must not be neglected. As usually made, even though locked and sealed, there is ample opportunity for the admission of dust. Refrigeration at the farm, down to 40° F. if possible, should be supplemented by conveyance in suitable railway vans. Unfortunately the railway companies have not yet seen their way to incur the expense of such vans. We are inclined to suggest that hospitals, when sufficiently close together, should combine. Now that the motor has arrived, it ought not to be difficult to find a satisfactory farm, within comparatively easy reach of London, from which the milk might be delivered by motor twice a day, in suitable receptacles, kept at a properly low temperature. We trust that the various hospital committees will not be content with the passage of mere resolutions, but that they will seriously attempt to put their milk supply on a proper hygienic basis.

